# Chronic Stress and Autoimmunity: The Role of HPA Axis and Cortisol Dysregulation

**DOI:** 10.3390/ijms26209994

**Published:** 2025-10-14

**Authors:** Sergio Gutierrez Nunez, Sara Peixoto Rabelo, Nikola Subotic, James Wilson Caruso, Nebojsa Nick Knezevic

**Affiliations:** 1 Department of Anesthesiology, Advocate Illinois Masonic Medical Center, Chicago, IL 60657, USA; sergio.gutierreznunez@aah.org (S.G.N.); sara.peixotorabelo@aah.org (S.P.R.); suboticnikola412@gmail.com (N.S.); wilcaruso@gmail.com (J.W.C.); 2 Department of Anesthesiology, University of Illinois, Chicago, IL 60612, USA; 3 Department of Surgery, University of Illinois, Chicago, IL 60612, USA

**Keywords:** autoimmune diseases, chronic stress, hypothalamic–pituitary–adrenal axis, cortisol, glucocorticoid receptor, immune dysregulation, neuroendocrine–immune interactions

## Abstract

Autoimmune diseases are chronic inflammatory conditions characterized by the breakdown of immune tolerance to self-antigens. While genetic and environmental factors play key roles, growing evidence highlights chronic stress as a significant contributor to immune dysregulation through its impact on the hypothalamic–pituitary–adrenal (HPA) axis. The HPA axis, primarily via cortisol secretion, serves as the major neuroendocrine mediator of stress responses, influencing both immune regulation and systemic homeostasis. This review synthesizes current literature on HPA axis physiology, the mechanisms of cortisol signaling, and the maladaptive effects of chronic stress. Emphasis is placed on clinical and experimental findings linking HPA dysfunction to immune imbalance and autoimmunity, as well as organ-specific consequences across neuroimmune, endocrine, cardiovascular, gastrointestinal, integumentary, and musculoskeletal systems. Chronic stress leads to impaired HPA axis feedback, glucocorticoid receptor resistance, and paradoxical cortisol dysregulation, fostering a pro-inflammatory state. This dysregulation promotes cytokine imbalance, weakens protective immune mechanisms, and shifts the immune response toward autoimmunity. Evidence from both human and animal studies associates persistent HPA dysfunction with diseases such as systemic lupus erythematosus, rheumatoid arthritis, and multiple sclerosis. HPA axis dysregulation under chronic stress constitutes a critical mechanistic link between psychological stress and autoimmune disease. Understanding these pathways provides opportunities for therapeutic interventions, including stress management, lifestyle modification, and neuroendocrine-targeted treatments. Future research should focus on multi-omics and longitudinal approaches to clarify the reversibility of HPA alterations and identify resilience factors.

## 1. Introduction

Autoimmune diseases constitute a group of systemic inflammatory conditions marked by immune system dysfunction that ultimately leads to the loss of tolerance toward the body’s own antigens [1]. Genetic, epigenetic, environmental, and hormonal factors may play a key role in the development of these conditions [2]. Among the hormonal and environmental factors, chronic psychological and physiological stress has emerged as an important modifier of immune regulation [3]. The hypothalamic–pituitary–adrenal (HPA) axis constitutes the principal neuroendocrine pathway through which stress alters immune responses, mainly via glucocorticoid secretion [4,5].

Stress-related mental and physiological inputs are integrated by limbic structures, particularly the amygdala and prefrontal cortex, which relay signals to the paraventricular nucleus (PVN) of the hypothalamus [4]. The PVN plays a pivotal role by secreting corticotropin-releasing hormone (CRH), the key initiator of the HPA axis cascade. CRH then stimulates the anterior pituitary to release adrenocorticotropic hormone (ACTH), ultimately driving cortisol secretion by the adrenal cortex [4,5].

Under physiological conditions, cortisol exerts many effects through complex mechanisms that include, but are not limited to, binding to intracellular glucocorticoid receptors (GRs) and regulating gene transcription [6]. It maintains homeostasis and exerts anti-inflammatory actions, such as regulating leukocyte trafficking and reducing pro-inflammatory cytokine secretion, via these pathways [7,8]. Moreover, cortisol enhances catecholamine actions, further shaping immune responses by modulating cytokine production [9].

However, under conditions of chronic stress, the HPA axis may lose its adaptive function. Prolonged cortisol exposure can lead to feedback impairment and glucocorticoid receptor resistance, resulting in diminished immunosuppressive effects and a pro-inflammatory state [10,11]. Prolonged stress may disturb cytokine regulation, promote sustained inflammatory activity, and weaken the numbers and functional capacity of protective immune cells [12]. Such chronic HPA axis dysfunction not only impacts the way the immune system responds to stressors but also shifts the immune environment toward autoimmunity, thereby contributing to the onset or exacerbation of diseases such as rheumatoid arthritis, lupus, or multiple sclerosis [13].

Clinical data shows elevated cortisol levels in patients with autoimmune diseases, supporting the link between chronic stress and autoimmunity [14]. Findings that may indicate that stress-induced neuroendocrine imbalance is not only linked to immune dysfunction but also clinically relevant in autoimmune disease pathogenesis. Accordingly, this review aims to summarize the physiology of the HPA axis and glucocorticoid signaling, analyze the mechanisms by which chronic stress induces immune dysregulation, examine organ-specific consequences across systems, and discuss therapeutic implications of targeting stress-responsive pathways in autoimmune disease.

## 2. The Physiology of the HPA Axis and Cortisol

The hypothalamic–pituitary–adrenal (HPA) axis is a neuroendocrine system composed of hormones, signaling pathways, and feedback loops that regulate various physiological processes [15]. One of the main functions of this axis is the response to stress, which begins in the paraventricular nucleus (PVN) of the hypothalamus with the release of corticotropin-releasing hormone (CRH). CRH further acts on the anterior pituitary gland to stimulate the secretion of adrenocorticotropic hormone (ACTH) [16]. ACTH then travels through the bloodstream to the adrenal cortex, specifically targeting the zona fasciculata to induce cortisol production [17]. This system is regulated by a negative feedback mechanism, in which both ACTH and cortisol act as inhibitors of CRH and ACTH secretion, thereby maintaining homeostasis [16,17].

Cortisol is a glucocorticoid hormone secreted at a basal state that follows a pulsatile and oscillatory nature according to the circadian rhythm [18]. Additionally, surges in cortisol levels occur in response to stressful stimuli. Neurons in the paraventricular nucleus (PVN) receive sensory input through several relays. One notable relay comes from the amygdala, a key brain structure involved in processing fear and emotions [4]. It communicates with the PVN via intermediary neurons in the bed nucleus of the stria terminalis and neighboring hypothalamic nuclei. This network ensures that emotional or psychological stressors can trigger an activation of the HPA axis and subsequent cortisol release [4,19].

**Figure 1:**
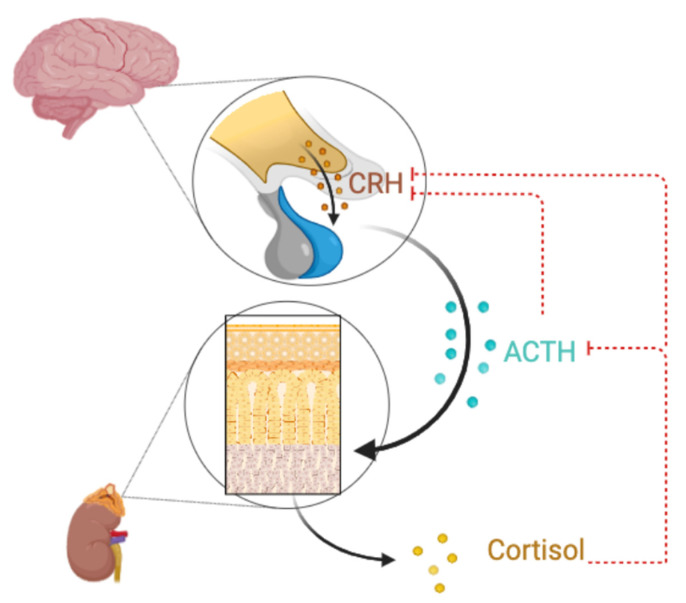
HPA axis showcasing the relationship between CRH, ACTH, and Cortisol. illustrating negative feedback from cortisol and ACTH on CRH production.

The physiological responses to cortisol depend on the specific organ or system involved. At the immune system level, anti-inflammatory properties of cortisol are widely known and used in clinical settings [17]. Cortisol can enhance innate immune system response, but it also reduces cytokine expression and production [20]. It acts through the glucocorticoid receptor (GR), which modulates gene expression by suppressing pro-inflammatory signaling pathways involving transcription factors such as NF-κB and AP-1 [18]. This results in decreased production of pro-inflammatory cytokines (including IL-1β, IL-6, and TNF-α), chemokines, and other immune-activating molecules. Moreover, these pro-inflammatory cytokines participate in a positive feedback loop that increases the stimulation of CRH and ACTH release [17]. As illustrated in Figure 1, hypothalamic CRH stimulates ACTH and downstream cortisol, while cortisol and ACTH close the loop by inhibiting CRH synthesis and release. Cortisol also inhibits the proliferation of T and B lymphocytes, limits leukocyte migration to inflamed tissues, and contributes to the maintenance of immune homeostasis [7,20].

Also, cortisol exerts a permissive effect, meaning that its presence enhances the action of other hormones such as catecholamines that are produced in response to central nervous system activation during the fight-or-flight reaction [9]. This interaction enables multiple physiological responses, including increased blood pressure and cardiac output, bronchodilation, or elevated blood glucose levels [6]. Once the stressor is removed, the HPA axis’s characteristic negative feedback loop acts to suppress cortisol secretion, restoring homeostasis. Altogether, this orchestrated system enables the body to mount an adequate response to stressful stimuli. In addition to their cardiovascular and metabolic effects, catecholamines also influence the immune system by modulating leukocyte trafficking and cytokine production, thereby contributing to an adequate immune system response [9].

Altogether, at the immune system level the HPA axis and cortisol maintain an immune balance under physiological conditions, predominantly by restraining inflammation. Catecholamines, released in parallel during stress and enhanced by cortisol presence, also contribute to this regulation by modulating leukocyte trafficking and cytokine production [9]. However, under conditions of prolonged stress, persistent elevation of cortisol can disrupt this regulatory system, leading to hypersecretion, sensitized stress responses, and adrenal exhaustion [16].

## 3. Chronic Stress and HPA Axis Dysregulation

The persistent activation of the HPA axis caused by multiple factors, including chronic stress, alters the normal regulatory processes, including loss of the normal pulsatile and circadian rhythms, impaired negative feedback mechanisms, and maladaptive changes in receptor sensitivity [10,11]. Over time, this can lead to adrenal hypertrophy and increased hormonal secretion, as demonstrated in animal models [19]. Excess cortisol can affect multiple structures, including key neuroanatomical sites such as the hippocampus and prefrontal cortex, where downregulation of glucocorticoid receptor (GR) density and impaired feedback inhibition further perpetuate HPA axis dysregulation [20].

Over time, the initial state of hypercortisolism may evolve into other stages: the extreme and persistent demand on the HPA axis present during chronic stress conditions may also lead to adrenal gland adaptive changes [11,21]. These alterations include a reduced responsiveness of the adrenal cortex to ACTH and other regulatory stimuli, resulting in a diminished capacity to produce adequate amounts of cortisol. This functional impairment is also referred to as adrenal resistance or adrenal exhaustion, due to the diminished responsiveness of the adrenal glands to regulatory stimuli [22]. Consequently, despite elevated or dysregulated circulating cortisol levels initially, a paradoxical hypocortisolism state may emerge in advanced or prolonged stress exposure due to this adrenal resistance and impaired signaling within the HPA axis [11,22].

The HPA axis alterations have many clinical implications: hypocortisolism and glucocorticoid resistance can impair multiple physiological systems and functions [6]. Additionally, these dysfunctions increase the vulnerability to inflammatory or autoimmune diseases due to inadequate glucocorticoid-mediated immune regulation [21].

## 4. Immunological Consequences of HPA Axis Dysfunction

The pulsatile, circadian, and natural rhythm of cortisol release plays a crucial role in synchronizing immune responses and maintaining homeostasis. A study that tested the primary hypothesis that chronic psychological stress impairs the immune system’s capacity to respond to the anti-inflammatory actions of glucocorticoid hormones. The results provided preliminary support: among parents of cancer patients, dexamethasone’s ability to suppress IL-6 production was significantly reduced compared to parents of medically healthy children [21]. These findings suggest that HPA axis dysregulation under chronic stress may lead to an altered cortisol response, impairing effective immune regulation. This may lead to sustained production of pro-inflammatory cytokines like IL-6 and TNF-α [23].

**Figure 2:**
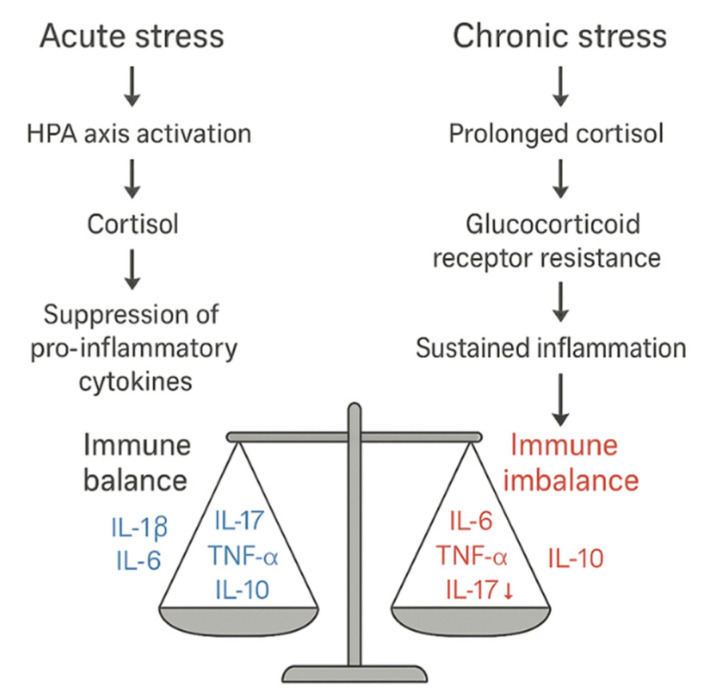
Acute vs. chronic stress: cytokine balance to imbalance via HPA axis dysregulation. Left: acute stress—cortisol restrains IL-1β/IL-6/TNF-α and supports IL-10, maintaining immune homeostasis. Right: chronic stress/GR resistance—↑ IL-6/TNF-α/IL-17 with ↓ IL-10 sustains inflammation and promotes autoimmunity. Abbreviations: ACTH, adrenocorticotropic hormone; CRH, corticotropin-releasing hormone; GR, glucocorticoid receptor; HPA, hypothalamic–pituitary–adrenal; IL, interleukin; MR, mineralocorticoid receptor; TNF-α, tumor necrosis factor-α.

Figure 2 contrasts acute versus chronic stress: cortisol restrains IL-1β/IL-6/TNF-α and supports IL-10 in the acute phase, whereas chronic HPA dysregulation/GR resistance tips the network toward ↑ IL-6/TNF-α/IL-17 and ↓ IL-10, favoring autoimmunity.

Additionally, excess cortisol may increase the binding to Mineralocorticoid Receptors that can produce a pro-inflammatory response [23,24]. When this happens at the level of certain immune cell populations, it has been associated with promoting pro-inflammatory responses [23]. Mineralocorticoid receptor (MR) activation in macrophages and other immune cells promotes upregulation of genes encoding cytokines like IL-6 and TNF-α [25].

Chronic HPA axis dysfunction and its impact on immune function have been linked to increased susceptibility to infections, delayed wound healing, greater risk for autoimmune conditions (e.g., rheumatoid arthritis, lupus), and even increased cardiovascular risk due to chronic inflammation [20,25]. One contributing factor may be that GC-mediated tolerogenic effects on dendritic cells are blunted during HPA axis dysfunction, reducing the capacity of the immune system to differentiate self from non-self [13,26]. As a result, a shift occurs from an environment of immune tolerance toward one that favors autoimmunity, which may be clinically manifested as the onset or exacerbation of autoimmune diseases, such as multiple sclerosis, rheumatoid arthritis, or lupus [13,26].

## 5. Clinical and Experimental Evidence

As previously mentioned, autoimmune diseases have a multifactorial etiology. In addition to environmental factors, numerous genetic, immunological, and hormonal components—many of which are independent of environmental conditions—play a significant role in their development. Physical and psychological stress are also considered important triggers in the onset of various autoimmune diseases [27].

Although many studies have not confirmed a strong role of genetic factors or predispositions in the development of autoimmune diseases, aside from the well-documented involvement of human leukocyte antigen (HLA) evidence for other genetic components, such as the regulatory gene CTLA-4, remains limited [28].

However, certain studies have indicated a significant influence of the PD-1 gene, a member of the CTLA-4 family, in the pathogenesis of more severe forms of autoimmune diseases, particularly rheumatic disorders [29,30,31]. In a study conducted by Criswell et al. [32], the association of the 620 V variant of the protein tyrosine phosphatase PTPN22 with several autoimmune diseases—including systemic lupus erythematosus (SLE), rheumatoid arthritis (RA), type 1 diabetes mellitus (T1DM), and Hashimoto’s thyroiditis—was examined, with a positive correlation observed. As summarized in Table 1, clinical and population studies consistently link chronic stress–related HPA axis dysregulation with elevated cortisol, pro-inflammatory signaling, and higher autoimmune disease risk.

**Table 1:** Clinical evidence linking chronic stress, HPA axis dysregulation, and autoimmunity.

Study/Authors	Population/Model	HPA Axis Findings	Cytokine/Immune Findings	Autoimmune/Clinical Outcome
Montero-López et al. (2017) [14]	35 women with SLE, Sjögren’s, systemic sclerosis vs. 30 controls	Elevated hair and salivary cortisol	Altered stress biomarkers; higher somatization and lower anxiety scores	Supports hyperactivation of the HPA axis in autoimmune disease
Song et al. (2018) [33]	Population-based cohort (>100,000) with stress-related disorders	Stress-related disorders are used as a proxy for chronic HPA dysregulation	Association with pro-inflammatory signaling pathways	Higher risk of autoimmune diseases (HR ≈ 1.1–1.5 by disease)
Zhang et al. (2025) [34]	Lupus-prone MRL-lpr vs. MRL/MPJ mice under predator stress	Heightened HPA sensitivity; stress reactivity increased	↑ IL-6, ↑ Th17, ↓ Treg; ↑ anti-dsDNA antibodies; proteinuria	Accelerated lupus progression in predisposed mice
Lei et al. (2025) [20]	Narrative mini-review—depressive disorders with chronic stress	HPA dysregulation; hippocampal GR downregulation/feedback impairment	Neuroinflammation; reduced glucocorticoid signaling efficacy	Increased vulnerability to inflammatory and autoimmune conditions
Hannibal & Bishop (2014) [24]	Clinical populations with chronic pain	Cortisol dysfunction in chronic stress contexts	Altered cytokine regulation and sensitization to pain	Rationale for stress-management to restore HPA–immune balance
Breunig et al. (2025) [27]	Genetic/psychiatric comorbidity analysis	Shared stress–immune regulatory architecture	Overlap of inflammatory pathways across disorders	Supports clustering of psychiatric and autoimmune diseases

Human cohorts and patient studies show elevated hair/salivary cortisol, altered stress biomarkers, and increased autoimmune risk; animal models demonstrate stress-exacerbated disease activity. Abbreviations: HPA, hypothalamic–pituitary–adrenal; HR, hazard ratio; SLE, systemic lupus erythematosus; Th17, T helper 17; Treg, regulatory T cell; ↑ increased; ↓ decreased

Interestingly, the same study reported a negative correlation between this genetic variant and the development of multiple sclerosis, a finding also confirmed by research conducted by Begovich et al. [35].

The development of autoimmune intestinal diseases, particularly Crohn’s disease, has been associated with specific genetic loci, including variants in the NOD2 and ATG16L1 genes. These genetic changes disrupt the autophagy process in dendritic cells of patients with Crohn’s disease [36]. Genome-wide association studies (GWAS) have significantly advanced our understanding of inflammatory bowel diseases (IBD), identifying 99 susceptibility loci—71 for Crohn’s disease and 47 for ulcerative colitis, with approximately one-third shared between the two. Shared risk genes are involved in immune regulation, particularly the IL-23/Th17 signaling pathway (e.g., STAT3, TYK2, IL23R, IL12B, JAK2), along with others such as REL, NKX2.3, SMAD3, IL10, CARD9, ICOSLG, ORMDL3, and PRDM1. Crohn’s disease is specifically associated with genes involved in autophagy and innate immunity (IRGM, ATG16L1, NOD2), highlighting defective bacterial clearance as a key mechanism. In contrast, ulcerative colitis shows associations with genes related to epithelial barrier function (LAMB1, GNA12, CDH1, HNF4A) [37].

Epigenetic mechanisms are increasingly recognized as key contributors to the pathogenesis of IBD, potentially explaining its “hidden heritability.” Epigenetics links environmental influences with genetic background by studying heritable changes in gene function that are not caused by alterations in the DNA sequence (e.g., mutations or recombination) [38]. The main epigenetic modifications include: [27] post-translational histone modifications, such as H3K27 acetylation; [28] expression of non-coding RNAs; and [29] DNA methylation, the most extensively studied to date [39].

A study, based on an animal model, conducted by Song et al. [33] explored the relationship between stress-related disorders and 41 autoimmune diseases in men and women using both population-based and sibling comparison designs. Risk levels varied by disease—for instance, hazard ratios ranged from 1.09 for rheumatoid arthritis to 1.49 for autoimmune thyroid disease, possibly reflecting differences in disease severity or underlying mechanisms [40]. An animal model study by Zhang et al. [34] explored the relationship between anxiety and systemic lupus erythematosus (SLE) in MRL-lpr and MRL/MPJ mice exposed to predator-induced stress (cats). MRL-lpr mice demonstrated heightened stress sensitivity, accompanied by pronounced immunological alterations—increased IL-6 levels, elevated anti-dsDNA antibodies, proteinuria, reduced Treg, and elevated Th17 cell proportions—indicative of accelerated disease progression. In contrast, MRL/MPJ mice exhibited a moderate inflammatory response without signs of immune dysregulation or renal impairment. These findings suggest that stress may exacerbate lupus pathology in genetically predisposed individuals.

A study by Montero Lopez et al. [14] examined HPA axis activity in 65 women, 35 with autoimmune diseases (SLE, Sjögren’s syndrome, systemic sclerosis) and 30 healthy controls. Higher cortisol levels were observed in both saliva and hair samples of the affected group, alongside elevated somatization scores and lower anxiety scores. Assessments included perceived stress measures, the SCL-90-R questionnaire, and cortisol analyses. These findings indicate increased short-term and long-term HPA axis activity in women with autoimmune diseases.

## 6. Molecular and Cellular Mechanisms

As mentioned before, the relation between stress and the immune system is complex and bidirectional. Glucocorticoids (GCs) are potent modulators of immune function. They affect trafficking, maturation, and function of dendritic cells, neutrophils, macrophages, and lymphocytes. GCs also influence cytokine production at both local and systemic levels. However, chronic or repeated stress exposure may lead to either immunosuppression or exaggerated inflammation, depending on the context and individual susceptibility [12]. Table 2 maps the cell-specific cytokine shifts under chronic stress—↑ TNF-α/IL-1β/IL-6 (macrophages), ↑ IL-17 (Th17), ↓ IL-10 and Treg function, and ↓ NK activity—collectively eroding immune tolerance.

**Table 2:** Immune cell-specific cytokine changes during chronic stress/HPA dysfunction.

Immune Cell Type	Normal (Acute Stress)	Chronic Stress/HPA Dysregulation	Key References
Macrophages	Balanced cytokine production; tissue repair support	↑ TNF-α, ↑ IL-1β, ↑ IL-6; pro-inflammatory phenotype	[23,25]
Dendritic Cells	Tolerogenic programming is maintained by cortisol; balanced antigen presentation	Reduced tolerogenicity and impaired maintenance of self-tolerance	[13,26]
T Regulatory Cells (Tregs)	↑ IL-10; suppression of autoreactive responses	↓ Treg number/function; ↓ IL-10; impaired tolerance	[12,21,34]
Th17 Cells	Limited activation under immune homeostasis	↑ IL-17; promotes autoimmunity and chronic inflammation	[20,34]
B Cells	Controlled antibody production	↑ Autoantibody production (e.g., anti-dsDNA)	[13,34]
Natural Killer (NK) Cells	Cytolytic activity and immune surveillance	↓ NK activity; reduced cytotoxicity and cytokine output	[41,42]
Microglia/Astrocytes	CNS homeostasis; controlled cytokine milieu	↑ Pro-inflammatory cytokines; impaired GR sensitivity; neuroinflammation	[20,43]

Chronic stress skews macrophages, dendritic cells, T/B cells, NK cells, and CNS glia toward pro-inflammatory phenotypes with reduced tolerance (↑ TNF-α/IL-1β/IL-6/IL-17; ↓ IL-10, ↓ NK function). Abbreviations: GR, glucocorticoid receptor; IFN-γ, interferon-γ; IL, interleukin; NK, natural killer; TNF-α, tumor necrosis factor-α.

Since the early hypothesis linking emotions and CNS activity to immune changes [44], research has revealed that stress-induced neuroendocrine mediators such as glucocorticoids and catecholamines affect immune cells at molecular, cellular, and systemic levels [3,45]. GCs generally suppress the production of pro-inflammatory cytokines like IL-1β, IL-6, and TNF-α, but prolonged stress impairs GC receptor signaling and may lead to glucocorticoid resistance, especially in the brain’s microglia and astrocytes [43].

Glucocorticoids (primarily cortisol in humans and corticosterone in rodents) have many permissive and stimulatory properties that help facilitate the peripheral actions of catecholamines [43]. Catecholamines modulate immunity via β-adrenergic receptors, favoring Th2-type responses and reducing Th1-mediated inflammation, which may contribute to increased autoimmunity or inadequate viral control related to chronic stress [41].

This glucocorticoid-driven modulation also extends to cytotoxic immune populations such as natural killer (NK) cells. Sustained glucocorticoid exposure has been shown to suppress NK cell activity, impacting their cytolytic functions and cytokine production. This leads to reduced immune surveillance and potentially increased autoantigen exposure [42].

In summary, chronic stress can affect immune function across multiple physiological systems, contributing to both acute and long-term health consequences. For instance, in individuals with chronic conditions such as type 2 diabetes, dysregulation of the hypothalamic–pituitary–adrenal (HPA) axis leads to reduced glucocorticoid sensitivity. This state contributes to elevated circulating pro-inflammatory cytokines, suggesting a mechanistic link between HPA axis dysfunction, and increased autoimmune and metabolic disease risk in chronically stressed populations [46].

The skin is another major target of stress-induced immune alterations; cytokine imbalance in this context—mostly IL6 and TNFa—can delay wound healing and accelerate cutaneous aging [47]. Furthermore, early-life stress has been identified as a critical factor in lifelong immune dysregulation. Individuals with adverse childhood experiences often exhibit elevated inflammatory markers even decades later, suggesting a sustained pro-inflammatory state [48].

Collectively, these findings underscore how chronic HPA axis activation and stress hormone imbalance contribute to immune dysfunction, promoting aging and increasing susceptibility to inflammatory, autoimmune, metabolic, and neuropsychiatric disorders [46].

### 6.1. Neuroimmune Consequences of HPA Axis Dysfunction in Chronic Stress and Autoimmunity

The interplay between the hypothalamic–pituitary–adrenal (HPA) axis and the nervous system is central to understanding how stress contributes to the pathogenesis and progression of autoimmune and neuropsychiatric disorders. Chronic psychological stress disrupts the regulation of the HPA axis, leading to sustained cortisol exposure, altered glucocorticoid receptor sensitivity, and downstream neuroimmune alterations [49]. These dysregulations have been implicated in the development of depressive phenotypes, particularly melancholic depression, where HPA axis hyperactivity and inflammation frequently coexist [50]. The “cortisol awakening response” (CAR), a biomarker of HPA axis integrity, has been linked not only to stress responsiveness but also to long-term neurological outcomes, with abnormal CAR patterns observed in various mood and cognitive disorders [51].

In animal and human models, stress-induced activation of the HPA axis leads to increased central cytokine production, microglial activation, and oxidative stress, which collectively impair neuroplasticity and promote neurodegeneration [43,52]. Prenatal stress further compounds this vulnerability, programming the offspring’s HPA axis toward hypersensitivity and increasing the lifetime risk of affective and inflammatory diseases [53]. These early-life perturbations may predispose individuals to altered emotional regulation, increased susceptibility to autoimmune conditions, and heightened neuroinflammatory responses to future stressors.

Moreover, aging and social isolation appear to exacerbate HPA dysregulation, creating a feedforward loop of immune senescence, neuroinflammation, and frailty, especially in neurodegenerative conditions such as Alzheimer’s disease [54]. In late-stage dementia, this crosstalk between the HPA axis and systemic immune responses has been shown to accelerate cognitive decline and impair behavioral resilience [55].

Taken together, these findings highlight the nervous system as both a mediator and a target of HPA axis dysfunction in the context of chronic stress and autoimmune vulnerability. Targeting neuroimmune pathways through both behavioral and pharmacologic means may represent a promising strategy to reduce neuropsychiatric comorbidities in autoimmune populations.

### 6.2. Endocrine Consequences of HPA Axis Dysfunction in Chronic Stress and Autoimmunity

HPA axis dysfunction disrupts endocrine balance and contributes to metabolic and autoimmune disorders. By regulating glucocorticoid release, it affects both energy metabolism and immune function, linking the neuroendocrine and peripheral systems. Agorastos et al. (2019) found that endocrine challenges trigger vagal changes, highlighting autonomic balance as key to HPA function [56]. This autonomic-endocrine interaction may be disrupted in chronic stress conditions, leading to maladaptive glucocorticoid secretion and enhanced inflammatory responses. Cadegiani and Kater (2019) observed impaired cortisol regulation and metabolic imbalance in athletes with overtraining syndrome (OTS), reflecting prolonged HPA hyperactivation [57]. These findings underscore the role of neuroendocrine stress pathways in systemic metabolic dysfunction. Prenatal factors can shape HPA-endocrine function. Xia et al. (2019) showed that prenatal ethanol exposure reprograms the HPA axis, increasing offspring susceptibility to metabolic syndrome by altering neuroendocrine-metabolic pathways [58]. Early-life stressors may thus predispose to metabolic and autoimmune disorders. Dysregulated insulin signaling is both a cause and effect of HPA axis hyperactivation in metabolic diseases. Cozma et al. (2022) linked impaired insulin pathways to chronic HPA activation, systemic inflammation, and metabolic dysfunction, highlighting endocrine-immune crosstalk in disease pathogenesis [59].

### 6.3. Cardiovascular Consequences of HPA Axis Dysfunction in Chronic Stress and Autoimmunity

Chronic HPA axis dysregulation is increasingly recognized as a key contributor to cardiovascular disease through its effects on cortisol, vascular tone, glucose metabolism, and inflammation. HPA axis hyperactivity has been linked to poor cardiovascular outcomes. Jokinen (2009) found that elevated HPA activity in mood disorder patients was associated with increased cardiovascular mortality, likely due to stress-related neuroendocrine imbalances and vascular dysfunction [60]. Girod and Brotman highlighted that chronic glucocorticoid imbalance contributes to hypertension, endothelial damage, dyslipidemia, and insulin resistance-key risk factors for cardiovascular disease [61]. Vodička et al. found that rats exposed to repeated stress had reduced hemodynamic and behavioral stability, suggesting excessive cortisol may increase vulnerability to stress-related cardiac events [62]. Joseph and Whirledge (2017) emphasize that chronic HPA activation disrupts reproductive and metabolic balance, promoting systemic inflammation and endothelial dysfunction—key contributors to cardiovascular risk [63]. This underscores HPA hyperactivity as a systemic driver of cardiovascular pathology under chronic stress.

### 6.4. Gastrointestinal Consequences of HPA Axis Dysfunction in Chronic Stress and Autoimmunity

The HPA axis regulates gastrointestinal motility, secretion, immune function, and gut–brain communication. Its dysregulation is linked to functional and inflammatory GI disorders and affects gut microbiota-CNS interaction. The brain–gut axis, comprising neural, hormonal, and immune pathways, is influenced by HPA-mediated stress. Jones et al. (2006) showed that abnormal HPA signaling causes visceral hypersensitivity, motility changes, and gut barrier dysfunction, contributing to functional GI disorders like IBS [64]. Kennedy et al. noted that IBS patients show prolonged HPA axis activation to acute stress, with sustained cortisol release and increased autonomic response. This hyperactivation may drive chronic visceral pain, motility changes, and inflammation, linking psychological stress to GI dysfunction [65]. Bonaz (2013) highlighted that in IBD, chronic stress and HPA dysregulation worsen intestinal inflammation through neuroendocrine-immune interactions. This interplay intensifies mucosal immune activation and disease flares, suggesting stress pathway modulation as a therapeutic target [66]. Emerging evidence shows that gut microbiota influences HPA axis regulation. Sandhu et al. (2017) noted that diet and microbiota affect the microbiota gut–brain axis, impacting neuropsychiatric health and HPA activity, because microbial metabolites are linked to heightened HPA responses and could worsen GI dysfunction and inflammation [67].

### 6.5. Tegumentar Consequences of HPA Axis Dysfunction in Chronic Stress and Autoimmunity

The skin functions as a peripheral neuroendocrine organ that mirrors the central HPA axis, expresses CRH, ACTH, and steroidogenic enzymes, allowing autonomous regulation of local stress responses, immune activity, and barrier integrity [68]. The cutaneous HPA-like axis is essential for maintaining local homeostasis and closely interacts with central neuroendocrine pathways. Jozic et al. highlight the dynamic cross-talk between the skin and the central HPA axis [69]. Dysregulation of the cutaneous HPA axis contributes to skin disorders and delayed wound healing. Vilela et al. showed that modulating HPA-driven inflammation improves wound repair by promoting tissue regeneration [70], highlighting the therapeutic potential of targeting HPA signaling in skin healing. Environmental stressors like UV radiation activate the HPA axis and worsen skin damage. Lim (2022) found that modulating HPA-related CYP11B activity can reduce early UV-induced collagen degradation, suggesting a glucocorticoid-mediated protective mechanism against photoaging [71]. Han et al. showed that UV skin irradiation triggers systemic HPA axis activation, affecting both skin health and reducing hippocampal neurogenesis and synaptic protein expression, revealing a bidirectional skin-brain stress axis [72]. Psychological stress affects skin barrier integrity and inflammation via HPA dysregulation. Lin et al. showed that stress-induced activation of 11β-HSD1 in keratinocytes raises local cortisol, impairing barrier repair and worsening atopic dermatitis [73]. The skin both regulates and is affected by HPA axis activity; stress-induced dysfunction worsens inflammation and healing, linking it to chronic inflammatory diseases.

### 6.6. Musculoskeletal Consequences of HPA Axis Dysfunction in Chronic Stress and Autoimmunity

The musculoskeletal system is strongly affected by HPA axis activity, with chronic glucocorticoid exposure leading to muscle wasting, pain, and changes in body composition [74]. Early-life stress sensitizes the HPA axis, causing persistent glucocorticoid receptor activation and sex-specific muscle pain differences. Green et al. found that females are more susceptible to HPA-driven chronic muscle pain [75]. Inflammation impacts muscle health through the HPA axis. Braun et al. showed that CNS inflammation activates the HPA axis, leading to muscle atrophy via catabolic pathways like ubiquitin-proteasome degradation [76]. This mechanism is key in cachexia and chronic inflammation, where sustained HPA activation speeds up muscle wasting [74]. Body composition and nutritional status affect HPA axis activity. Gonsalves et al. showed that lean mass and fat levels influence cortisol secretion and feedback, suggesting muscle mass may buffer against HPA hyperactivity [77]. Pavlou et al. found that micronutrient deficiencies (e.g., magnesium, vitamin D) worsen stress-related muscle tension and bruxism via enhanced glucocorticoid signaling and neuromuscular impairment [78]. Collectively, these findings indicate that HPA axis dysregulation contributes to muscle atrophy, pain syndromes, and metabolic alterations.

### 6.7. Hematopoietic Consequences of HPA Axis Dysfunction in Chronic Stress and Autoimmunity

Chronic stress profoundly alters the hematopoietic system through dysregulation of the hypothalamic–pituitary–adrenal (HPA) axis and the sympathetic nervous system. Elevated cortisol and catecholamines act on bone marrow, leading to an expansion of myeloid lineages at the expense of lymphoid progenitors, thereby creating a microenvironment that favors autoimmunity [79]. Heidt et al. demonstrated that chronic variable stress activates hematopoietic stem cells (HSCs), driving sustained myelopoiesis and enhanced release of inflammatory monocytes into circulation [80]. This shift not only promotes systemic inflammation but also provides a mechanistic link between stress and the exacerbation of autoimmune pathology.

Hofmann et al. and Cordeiro Gomes et al. highlighted that the bone marrow niche is highly adaptive to acute stress and infection, but chronic exposure leads to maladaptive remodeling that shifts differentiation toward inflammatory phenotypes [81,82]. Such changes disrupt the balance of self-renewal and differentiation, impairing the regenerative capacity of HSCs. Moreover, Bogeska et al. showed that inflammatory exposure induces long-term impairment of HSC self-renewal and accelerates their functional aging, contributing to persistent immune dysregulation [83].

On the other hand, immune–adrenal cross-talk also plays a central role in regulating hematopoietic activity. Fudulu et al. demonstrated that monocytes interact directly with adrenal zona fasciculata cells, shaping glucocorticoid output and thereby closing a feedback loop between hematopoiesis and HPA function [84]. Such bidirectional communication suggests that stress-induced alterations in hematopoietic output can further reinforce maladaptive HPA activity, perpetuating a cycle of inflammation.

Metabolic and nutritional stressors also intersect with HPA-mediated hematopoietic changes. Zhou et al. reported that bone marrow immune cells sense nutritional fluctuations to regulate systemic energy balance, while Horikawa et al. found that chronic stress alters lipid mediator profiles and gene expression in bone marrow and spleen, reshaping immune cell composition [85,86]. Together, these findings support that both endocrine and metabolic stressors converge on the bone marrow to promote a pro-inflammatory hematopoietic bias.

**Figure 3:**
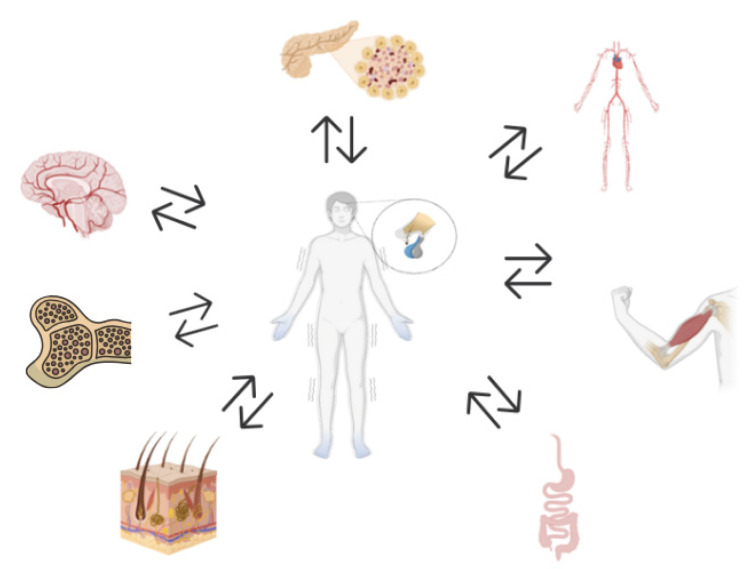
HPA axis dysregulation leads to systemic pathology affecting various systems, including the brain, musculoskeletal, gastrointestinal, integumentary, hematopoietic and cardiovascular system.

As shown in Figure 3, chronic HPA axis dysregulation—loss of circadian cortisol rhythm and impaired feedback—drives systemic pathology across the brain, musculoskeletal, gastrointestinal, integumentary, cardiovascular and hematopoietic systems. The interplay between inflammation, nutrition, and glucocorticoid receptor sensitivity emphasizes the importance of targeting HPA activity for the prevention and treatment of muscle-related complications in autoimmune and chronic stress conditions.

## 7. Therapeutic Implications and Interventions

Given the broad and systemic impact of chronic stress and HPA axis dysregulation there is growing interest in non-pharmacological strategies to mitigate these effects. Evidence supports the role of mindfulness and nature-based interventions in reducing work-related stress and promoting psychological resilience. Mindfulness-based practices, in particular, have been shown to enhance emotional regulation and downregulate physiological stress responses, contributing to better HPA axis balance and overall well-being [87]. Similarly, increased exposure to natural environments appears to lower cortisol levels and improve mental health outcomes, suggesting a potential role in restoring neuroendocrine homeostasis [87].

In clinical populations, such as cancer survivors, adaptive coping strategies are directly associated with improved quality of life and reduced psychological burden [88]. Among frontline healthcare workers, especially nurses during the COVID-19 pandemic, effective stress management approaches, including social support, emotion-focused coping, and problem-solving—were critical to reducing burnout and preserving mental health [89]. Moreover, in animal models, gradual exposure to stressors and social buffering, as demonstrated in piglets through pre-weaning socialization, reduced physiological stress markers, highlighting the value of structured environmental and social interventions in modulating stress responses [90].

Lifestyle interventions also play a key role in long-term stress management and prevention of HPA axis maladaptation. In individuals with chronic conditions such as hypertension, adherence to lifestyle reform—including diet, exercise, and stress reduction techniques—has been associated with improved physiological parameters and better disease control [91].

Non-pharmacological interventions have also gained growing recognition as essential adjuncts in the management of autoimmune diseases. These strategies address not only the physical symptoms but also the psychological and functional impairments often experienced by patients. Mind–body practices such as meditation and mindfulness-based stress reduction (MBSR) have demonstrated positive effects on stress perception and disease-related outcomes. In a pilot randomized controlled trial, an adapted MBSR protocol for patients with systemic lupus erythematosus (SLE) led to improved emotional coping and decreased psychological distress [92]. Meditation has also been shown to modulate the inflammatory response and reduce pain, possibly through neuroendocrine and immune system interactions [93]. Similarly, wellness practices—including stress management, regular physical activity, and adequate sleep—have been advocated as core components of holistic care in rheumatoid arthritis (RA), with potential benefits extending beyond conventional treat-to-target strategies [94].

Physical activity plays a central role in many non-pharmacological protocols, with evidence supporting its impact on fatigue, pain, and physical functioning. For instance, fatigue in SLE has been directly associated with reduced physical fitness, suggesting a target for tailored exercise interventions [95]. In patients with ankylosing spondylitis, a randomized controlled trial of tele-yoga during the COVID-19 pandemic demonstrated meaningful improvements in disease activity and quality of life compared to controls [96]. These findings are consistent with broader evidence supporting exercise-based therapies in autoimmune-related chronic pain conditions [93]. Expert consensus, including EULAR recommendations, emphasizes a multidisciplinary approach for systemic lupus erythematosus and systemic sclerosis, highlighting the importance of patient education, psychosocial support, and physical therapy in routine care [97]. However, gaps in research remain—particularly in low-resource settings—underscoring the need for high-quality studies to better define and implement effective non-pharmacological strategies globally [98].

## 8. Future Directions and Gaps

Despite the advances in understanding the relationship between HPA axis dysfunction, immune regulation, and autoimmunity, many questions remain unanswered. Some gaps include the precise molecular mechanisms driving the transition from immune tolerance to autoimmunity under chronic stress, the temporal dynamics of glucocorticoid resistance and hypercortisolism, and the specific factors that determine individual susceptibility or resilience to HPA axis dysregulation [13]. Moreover, the development of autoimmunity is multifactorial: genetic susceptibility, epigenetic modifications, environmental exposures, the microbiome, sex hormones, alongside neuroendocrine stress pathways may interact and contribute to the pathogenesis of autoimmunity [2,99].

Another critical area requiring clarification is the reversibility of HPA axis alterations and immune dysfunction once chronic stress or inflammation is resolved. Additionally, the role of circadian rhythm disturbances in modulating both endocrine and immune functions, as well as their clinical significance in humans, warrants further exploration [100]. Addressing these issues will require longitudinal, multi-omics, systems-level approaches and translational studies integrating clinical, molecular and behavioral endpoints.

## Data Availability

No new data were created or analyzed in this study.

## Abbreviations

The following abbreviations are used in this manuscript:

11β-HSD1	11β-hydroxysteroid dehydrogenase 1;
ACTH	Adrenocorticotropic Hormone
AP-1	Activator Protein-1
ATG16L1	Autophagy Related 16 Like 1
CARD9	Caspase Recruitment Domain Family Member 9
CAR	Cortisol Awakening Response
CDH1	Cadherin 1
COVID-19	Coronavirus Disease 2019
CRH	Corticotropin-Releasing Hormone
CTLA-4	Cytotoxic T-Lymphocyte Associated Protein 4
CYP11B	Cytochrome P450 Family 11 Subfamily B
DNA	Deoxyribonucleic Acid
dsDNA	Double-Stranded Deoxyribonucleic Acid
EULAR	European League Against Rheumatism
GC	Glucocorticoid
GI	Gastrointestinal
GNA12	Guanine nucleotide-binding protein subunit alpha-12
GR	Glucocorticoid Receptor
GWAS	Genome-Wide Association Studies
H3K27	Histone H3 Lysine 27
HLA	Human Leukocyte Antigen
HNF4A	Hepatocyte Nuclear Factor 4 Alpha
HPA axis	Hypothalamic–Pituitary–Adrenal Axis
HR	Hazard Ratio
HSC	hematopoietic stem cells
ICOSLG	Inducible T Cell Costimulator Ligand
IFN-γ	Interferon Gamma
IL	Interleukin
IL1B	Interleukin 1 Beta
IL6	Interleukin 6
IL10	Interleukin 10
IL12B	Interleukin 12 Beta
IL23R	Interleukin 23 Receptor
IBD	Inflammatory Bowel Diseases
IRGM	Immunity-Related GTPase Family M Protein
JAK2	Janus Kinase 2
LAMB	Laminin Subunit Beta 1
MBSR	Mindfulness-Based Stress Reduction
MR	Mineralocorticoid Receptor
mRNA	Messenger Ribonucleic Acid
MRL/MPJ	Mouse Strain Control for MRL-lpr
MS	Multiple Sclerosis
NF-κB	Nuclear Factor Kappa-Light-Chain-Enhancer of Activated B Cells
NK cells	Natural Killer Cells
NKX2.3	NK2 Homeobox 3
NOD2	Nucleotide-Binding Oligomerization Domain Containing 2
OTS	Overtraining Syndrome
ORMDL3	ORMDL Sphingolipid Biosynthesis Regulator 3
PD-1	Programmed Cell Death Protein 1
PTPN22	Protein Tyrosine Phosphatase Non-Receptor Type 22
PRDM1	PR/SET Domain 1
PVN	Paraventricular Nucleus
RA	Rheumatoid Arthritis
REL	Proto-Oncogene, NF-κB Subunit
SCL-90-R	Symptom Checklist-90-Revised
SLE	Systemic Lupus Erythematosus
SMAD3	Mothers Against Decapentaplegic Homolog 3
STAT3	Signal Transducer and Activator of Transcription 3
T1DM	Type 1 Diabetes Mellitus
TGF-β	Transforming Growth Factor Beta
Th1	T Helper Cell Subset 1
Th2	T Helper Cell Subset 2
Th17	T Helper Cell Subset 17
TLA	Three letter acronyms
TLR	Toll-Like Receptor
TNF-α	Tumor Necrosis Factor Alpha
TNFa	Tumor Necrosis Factor Alpha (gene symbol)
Tregs	Regulatory T Cells
TYK2	Tyrosine Kinase 2
UV	Ultraviolet
